# Low baseline alanine aminotransferase is associated with primary non-response to infliximab induction in pediatric Crohn's disease: a retrospective cohort study

**DOI:** 10.3389/fped.2026.1948999

**Published:** 2026-09-25

**Authors:** Yuhang Wang, Cheng Guo, Jie Wu

**Affiliations:** Gastroenterology, Hepatology and Nutrition Center, Beijing Children's Hospital, Capital Medical University, National Center for Children's Health, Beijing, China

**Keywords:** alanine aminotransferase, biologic therapy, inflammatory bowel disease, infliximab, pediatric Crohn's disease, primary non-response

## Abstract

**Background:**

Low alanine aminotransferase (ALT) has been associated with unfavorable inflammatory and nutritional phenotypes, but its relationship with infliximab (IFX) response in pediatric Crohn's disease (CD) is uncertain.

**Methods:**

We retrospectively studied 116 children with CD who received IFX induction at weeks 0, 2, and 6 and underwent response assessment at approximately week 14. Low ALT was defined as <10 U/L using a rounded, data-informed working threshold. Primary non-response (PNR) required concurrent clinical non-response, biochemical activity, and objective endoscopic or cross-sectional imaging evidence and was independently adjudicated by two pediatric gastroenterologists blinded to baseline ALT. Firth logistic regression assessed the association between low ALT and PNR after adjustment for baseline SES-CD, IFX dose, and concomitant immunomodulator use.

**Results:**

Fifty-eight children (50.0%) had low ALT and 21 (18.1%) had PNR. PNR occurred more often with low ALT (27.6% vs. 8.6%; *P* = 0.008). Low ALT remained associated with PNR after adjustment (OR, 4.03; 95% CI, 1.48–12.65; *P* = 0.006), and each 5-U/L decrease in ALT was associated with higher odds of PNR (OR, 1.50; 95% CI, 1.04–2.59; *P* = 0.022). Discrimination was limited (AUC, 0.677; bootstrap 95% CI, 0.547–0.802).

**Conclusion:**

Low baseline ALT was associated with PNR to IFX in pediatric CD. This exploratory association and the 10-U/L working threshold require external validation and should not guide treatment decisions in isolation.

## Introduction

Pediatric inflammatory bowel disease (IBD) is increasingly recognized worldwide, and Crohn's disease (CD) may impair growth, nutritional status, psychosocial well-being, and long-term quality of life (1–4). Infliximab (IFX) is an established treatment for children with moderate-to-severe or refractory CD (5, 6). Nevertheless, some children do not achieve satisfactory disease control during induction and are classified as having primary non-response (PNR) (7). Because PNR may prompt dose optimization, treatment escalation, or a change in biologic therapy, readily available markers that provide complementary information about a child's baseline condition are clinically relevant.

Alanine aminotransferase (ALT) is routinely used to identify hepatocellular injury, but a low value should not automatically be interpreted as evidence of optimal health. In older adults and several chronic disease populations, low ALT has been associated with frailty, sarcopenia, and adverse outcomes (8–14). Although these observations do not establish a specific mechanism, they suggest that low ALT may sometimes reflect diminished metabolic or musculoskeletal reserve rather than simply the absence of liver injury.

Evidence in IBD remains limited. A nationwide adult study found that low ALT was associated with higher C-reactive protein and fecal calprotectin levels and lower hemoglobin and albumin levels (15). In a pediatric IBD cohort, low transaminase levels were associated with lower hemoglobin, albumin, and folate levels and with unfavorable clinical outcomes during follow-up (16). However, that study included children with both ulcerative colitis and CD and did not specifically evaluate response to biologic induction. The relevance of low ALT to early IFX outcomes in pediatric CD therefore remains uncertain.

Children with CD are particularly susceptible to chronic inflammation, reduced intake, altered body composition, protein-energy malnutrition, and growth impairment (17–20). Low ALT may therefore identify an unfavorable inflammatory and nutritional phenotype, although it is not a direct measure of nutritional status, muscle mass, or IFX pharmacokinetics. Thresholds used in previous IBD studies have also differed, including ALT <5 U/L in a pediatric cohort (16) and ALT <10 U/L in a large adult cohort (15); no externally validated threshold is available for pediatric CD.

This study aimed to characterize the prevalence and baseline clinical phenotype of low ALT in children with CD receiving IFX and to evaluate its association with rigorously adjudicated PNR after adjustment for selected disease-severity and treatment-related factors. ALT <10 U/L was used as a rounded, data-informed working threshold after an exploratory cohort analysis yielded a cut-point near 10 U/L; its use was also supported by clinical interpretability and prior adult IBD literature. Continuous ALT and exploratory receiver operating characteristic (ROC) analyses were used to assess the robustness and discriminatory context of this threshold, not to develop or validate a clinical prediction model.

## Method

### Ethics statement

This study was reviewed and approved by the Ethics Committee of Beijing Children's Hospital, Capital Medical University [approval number: (2026)-E-066-R]. The requirement for written informed consent was waived because the study retrospectively used de-identified data collected during routine care.

### Study design and participants

This single-center retrospective cohort study was conducted at Beijing Children's Hospital, Capital Medical University, and is reported in accordance with the Strengthening the Reporting of Observational Studies in Epidemiology (STROBE) recommendations (21). We reviewed the medical records of children with CD who received IFX between January 2017 and November 2024. No *a priori* sample-size calculation was performed; all eligible patients available in the analytical database during the study period were included.

Patients were eligible if they were younger than 18 years, fulfilled the revised Porto criteria for pediatric CD (22), received a three-dose IFX induction regimen at weeks 0, 2, and 6, with actual doses of 5–10 mg/kg (5, 6), and had an evaluable post-induction assessment at approximately week 14, coinciding with the first planned maintenance infusion. Exclusion criteria were interruption of the induction regimen, absence of an evaluable primary exposure or outcome, severe liver disease, inborn errors of metabolism, malignancy, or another condition considered likely to substantially alter ALT. The final analytical cohort comprised 116 children.

Eligible patients were identified retrospectively from a study database that had been constructed to contain only patients meeting the eligibility criteria rather than all children initially assessed for IFX initiation. Consequently, the total number screened and the numbers and reasons for exclusion were not retrievable, and no retrospective participant-flow counts were reconstructed.

### Data collection and definitions

Baseline was defined as the clinical or laboratory assessment closest to the first IFX infusion within the preceding 4 weeks. Demographic and clinical variables included age, sex, disease duration, height, weight, body mass index (BMI), growth failure, nutritional support, previous biologic exposure, and concomitant corticosteroid and immunomodulator treatment. BMI was calculated as weight in kilograms divided by height in meters squared. Disease location, upper gastrointestinal involvement, disease behavior, and perianal disease were classified according to the Paris classification (23). Disease activity was assessed using the Pediatric Crohn's Disease Activity Index (PCDAI) (24), and endoscopic severity was assessed using the Simple Endoscopic Score for Crohn's Disease (SES-CD) (25). IFX induction dose was expressed in mg/kg; absolute doses were divided by baseline weight, whereas verified mg/kg values were retained when the absolute dose was unavailable.

Laboratory variables included ALT, hemoglobin, white blood cell count, neutrophil count, lymphocyte count, monocyte count, platelet count, C-reactive protein, erythrocyte sedimentation rate, albumin, prealbumin, and fecal calprotectin (FC). FC values of 0 were retained as actual measured values. Results reported as >1800 were treated as upper-limit-censored observations and were summarized categorically rather than assigned an exact numeric value.

The primary exposure was low baseline ALT, defined as ALT <10 U/L. An exploratory ROC analysis in the same cohort yielded a pROC Youden threshold of 9.9 U/L; this value was rounded to 10 U/L for interpretability and was consistent with the threshold used in a large adult IBD study (15). Because the threshold was informed by the study cohort and has not been externally validated, it was treated as a working classification rule rather than a biological reference limit or treatment-decision boundary. The earlier pediatric IBD study used ALT <5 U/L (16), further underscoring the absence of a standardized pediatric definition. Children with ALT ≥10 U/L formed the comparison group.

Post-induction response was assessed at approximately week 14 after the standard three-dose IFX induction regimen administered at weeks 0, 2, and 6. Two senior pediatric gastroenterologists independently reviewed the medical records and were blinded to baseline ALT results. Disagreements were resolved by consensus.

PNR required all of the following: (1) clinical non-response, defined as a PCDAI >10 with a decrease of <15 points from baseline; (2) at least one biochemical criterion, defined as C-reactive protein persistently >10 mg/L with a decrease of <50% from baseline or fecal calprotectin >250 μg/g; and (3) objective evidence of active inflammation at approximately week 14. Objective evidence comprised endoscopic non-response, defined as failure to achieve a ≥ 50% reduction in SES-CD, or persistent active inflammation on intestinal ultrasound or magnetic resonance enterography. Infection, treatment intolerance, poor adherence, and structural complications judged to be the primary explanation for symptoms were excluded before PNR classification. All 21 children classified as having PNR met the objective endoscopic or cross-sectional imaging criterion. Children who did not meet the composite PNR definition were classified as responders.

### Statistical analysis

Analyses were conducted using R version 4.5.2 (R Foundation for Statistical Computing, Vienna, Austria). Continuous variables were summarized as medians and interquartile ranges and compared using the Mann–Whitney U test. Categorical variables were summarized as counts and percentages and compared using the Pearson chi-square test or Fisher exact test, as appropriate. Sparse multicategory tables were evaluated using a Monte Carlo conditional test based on 200,000 random tables. *P* values in the baseline comparison tables were unadjusted and interpreted descriptively. Variables included in the primary and sensitivity models were complete; no missing-data imputation was performed.

Given the limited number of PNR events and the presence of sparse phenotype categories, Firth logistic regression was used for the primary adjusted analysis with the logistf package (26). Low ALT was the exposure of interest, and the primary model adjusted for baseline SES-CD, actual IFX dose in mg/kg, and concomitant immunomodulator use. These covariates were selected on clinical grounds to represent endoscopic severity and treatment exposure rather than through screening based on univariable *P* values. Results are reported as ORs, profile penalized-likelihood 95% CIs, and penalized likelihood-ratio *P* values.

Sensitivity analyses modeled ALT continuously per 5-U/L decrease using the primary covariates; repeated the primary model after excluding patients with ALT >40 U/L; added age and sex to the primary model; and evaluated alternative clinical domains. The phenotype-domain model included L4b involvement, complicated behavior (B2, B3, or B2B3), and perianal disease. An additional model added L4b to the primary covariates, and an inflammation-nutrition model included SES-CD, C-reactive protein, and albumin. The primary model was also refitted using conventional maximum-likelihood logistic regression. Internal stability of the primary Firth coefficient was evaluated using 2,000 ordinary nonparametric bootstrap resamples of the full cohort. Bootstrap summaries included the median OR, percentile range, and proportion of estimates >1; these analyses were not interpreted as external validation.

Exploratory discrimination of continuous ALT for PNR was summarized using the AUC with the pROC package. A 2,000-replicate stratified bootstrap was used for the AUC 95% CI. The empirical Youden threshold, sensitivity, and specificity were calculated in the full cohort. Threshold stability was evaluated separately using 5,000 ordinary nonparametric bootstrap resamples of the full cohort, with the Youden threshold recalculated in each resample. All *P* values were two-sided. No multiplicity adjustment was applied; interpretation emphasized effect estimates, CIs, and consistency across analyses.

OpenAI ChatGPT (version/access context: ChatGPT web application accessed in 2026; models: GPT-5.6 Thinking and GPT-5.6 Sol; source: OpenAI, San Francisco, CA, United States) was used to assist with manuscript drafting and language editing, R-code drafting, and figure layout. It was not used for outcome adjudication or to independently analyze or interpret source data. All code was executed by the authors, and all numerical results, references, and visualizations were checked against the source data.

## Results

### Study population

A total of 116 children with Crohn's disease were included. Fifty-eight (50.0%) had baseline ALT <10 U/L, and 21 (18.1%) were classified as having PNR at approximately week 14. All 21 children with PNR had objective evidence of active inflammation on endoscopy, intestinal ultrasound, or magnetic resonance enterography. Variables required for all reported multivariable models were complete.

### Baseline characteristics according to ALT status

Selected baseline characteristics according to ALT status are presented in Table 1; the complete comparison is provided in Supplementary Table S2. Compared with children with ALT ≥10 U/L, those with low ALT had higher C-reactive protein [8.00 (5.00, 39.00) vs. 5.00 (4.00, 9.50) mg/L; *P* = 0.019] and erythrocyte sedimentation rate [27.00 (14.25, 43.25) vs. 13.50 (7.25, 34.50) mm/h; *P* = 0.014], together with lower hemoglobin [105.00 (98.00, 114.00) vs. 111.00 (102.25, 128.75) g/L; *P* = 0.008], albumin [36.30 (32.25, 38.50) vs. 38.35 (34.90, 41.50) g/L; *P* = 0.002], and prealbumin [127.50 (98.00, 169.50) vs. 151.00 (116.25, 215.75) mg/L; *P* = 0.007].

**Table 1:** Selected baseline characteristics of children with Crohn's disease according to baseline ALT status.

Variable	Total (*n* = 116)	Non-low ALT (*n* = 58)	Low ALT (*n* = 58)	*P* value
Demographic and anthropometric characteristics				
Male sex, *n* (%)	72 (62.1)	40 (69.0)	32 (55.2)	0.126
Age, years	12.0 (9.0, 13.5)	11.8 (9.8, 13.5)	12.1 (8.6, 13.5)	0.799
BMI, kg/m^2^	15.12 (13.78, 17.21)	15.09 (13.59, 18.23)	15.45 (13.90, 17.04)	0.903
Disease activity and growth				
Growth failure, *n* (%)	39 (33.6)	19 (32.8)	20 (34.5)	0.844
PCDAI	30.00 (15.00, 38.12)	28.50 (15.62, 36.88)	32.00 (15.62, 40.00)	0.484
SES-CD	6.00 (4.00, 11.00)	6.00 (4.00, 11.00)	7.00 (4.00, 10.00)	0.772
Laboratory characteristics				
CRP, mg/L	6.50 (4.00, 17.75)	5.00 (4.00, 9.50)	8.00 (5.00, 39.00)	0.019
ESR, mm/h	21.00 (11.00, 39.25)	13.50 (7.25, 34.50)	27.00 (14.25, 43.25)	0.014
Hemoglobin, g/L	108.00 (99.00, 122.00)	111.00 (102.25, 128.75)	105.00 (98.00, 114.00)	0.008
Albumin, g/L	37.60 (33.77, 40.42)	38.35 (34.90, 41.50)	36.30 (32.25, 38.50)	0.002
Prealbumin, mg/L	140.00 (107.50, 195.50)	151.00 (116.25, 215.75)	127.50 (98.00, 169.50)	0.007
Treatment characteristics				
Prior biologic exposure, *n* (%)	7 (6.0)	3 (5.2)	4 (6.9)	1.000
Concomitant immunomodulator use, *n* (%)	41 (35.3)	20 (34.5)	21 (36.2)	0.846
Concomitant corticosteroid use, *n* (%)	29 (25.0)	14 (24.1)	15 (25.9)	0.830
IFX dose, mg/kg	7.46 (6.48, 8.51)	7.58 (6.81, 8.65)	7.26 (6.30, 8.32)	0.171
Induction outcome				
Primary non-response to IFX, *n* (%)	21 (18.1)	5 (8.6)	16 (27.6)	0.008

Data are presented as median (Q1, Q3) or *n* (%). *P* values are unadjusted and should be interpreted descriptively. Continuous variables were compared using the Mann–Whitney U test; categorical variables were compared using the Pearson chi-square test or Fisher exact test, as appropriate. The complete baseline comparison is provided in Supplementary Table S2. ALT, alanine aminotransferase; BMI, body mass index; CRP, C-reactive protein; ESR, erythrocyte sedimentation rate; IFX, infliximab; PCDAI, Pediatric Crohn's Disease Activity Index; PNR, primary non-response; SES-CD, Simple Endoscopic Score for Crohn's disease.

No clear between-group differences were observed in age, sex, body mass index, Paris disease location or behavior, perianal disease, growth failure, Pediatric Crohn's Disease Activity Index, Simple Endoscopic Score for Crohn's Disease (SES-CD), nutritional support, actual infliximab dose, or concomitant immunomodulator or corticosteroid use. PNR occurred more often in the low-ALT group than in the non-low-ALT group (27.6% vs. 8.6%; *P* = 0.008).

### Baseline characteristics according to infliximab response

Selected baseline characteristics according to infliximab response are shown in Table 2; the complete comparison is provided in Supplementary Table S3. Children with PNR had lower baseline ALT than responders [8.40 (5.80, 9.70) vs. 11.20 (8.50, 18.50) U/L; *P* = 0.012], and low ALT was more frequent in the PNR group (76.2% vs. 44.2%; *P* = 0.008). White blood cell, neutrophil, and monocyte counts were also higher among children with PNR (*P* = 0.045, *P* = 0.016, and *P* = 0.017, respectively). No clear between-group difference in BMI was observed [PNR, 14.53 (12.91, 16.41) vs. responders, 15.36 (13.98, 17.45) kg/m^2^; *P* = 0.132].

**Table 2:** Selected baseline characteristics of children with Crohn's disease according to primary response to infliximab induction.

Variable	Total (*n* = 116)	Response (*n* = 95)	Primary non-response (*n* = 21)	*P* value
Demographic and anthropometric characteristics				
Male sex, *n* (%)	72 (62.1)	59 (62.1)	13 (61.9)	0.986
Age, years	12.0 (9.0, 13.5)	12.1 (9.8, 13.5)	11.6 (5.3, 12.6)	0.101
BMI, kg/m^2^	15.12 (13.78, 17.21)	15.36 (13.98, 17.45)	14.53 (12.91, 16.41)	0.132
Disease phenotype and activity				
Upper gastrointestinal modifier, *n* (%)				0.004
None	77 (66.4)	65 (68.4)	12 (57.1)	
L4a	34 (29.3)	29 (30.5)	5 (23.8)	
L4b	5 (4.3)	1 (1.1)	4 (19.0)	
Perianal disease, *n* (%)	23 (19.8)	18 (18.9)	5 (23.8)	0.562
PCDAI	30.00 (15.00, 38.12)	30.00 (16.25, 36.25)	30.00 (15.00, 40.00)	0.782
SES-CD	6.00 (4.00, 11.00)	6.00 (4.00, 11.00)	6.00 (5.00, 12.00)	0.675
Laboratory characteristics				
ALT, U/L	9.90 (7.70, 18.10)	11.20 (8.50, 18.50)	8.40 (5.80, 9.70)	0.012
Low ALT (<10 U/L), *n* (%)	58 (50.0)	42 (44.2)	16 (76.2)	0.008
CRP, mg/L	6.50 (4.00, 17.75)	6.00 (4.00, 13.50)	8.00 (5.00, 39.00)	0.123
WBC, × 10⁹/L	7.78 (5.74, 10.29)	7.23 (5.63, 9.13)	9.89 (6.68, 13.80)	0.045
Neutrophils, × 10⁹/L	4.63 (3.26, 6.12)	4.21 (3.12, 5.72)	5.42 (4.16, 8.37)	0.016
Monocytes, × 10⁹/L	0.51 (0.38, 0.83)	0.49 (0.36, 0.78)	0.72 (0.53, 0.99)	0.017
Albumin, g/L	37.60 (33.77, 40.42)	37.60 (34.20, 40.45)	38.00 (32.70, 39.10)	0.680
Prealbumin, mg/L	140.00 (107.50, 195.50)	144.00 (108.00, 200.50)	124.00 (105.00, 195.00)	0.616
Treatment characteristics				
Concomitant immunomodulator use, *n* (%)	41 (35.3)	34 (35.8)	7 (33.3)	0.831
Concomitant corticosteroid use, *n* (%)	29 (25.0)	22 (23.2)	7 (33.3)	0.330
IFX dose, mg/kg	7.46 (6.48, 8.51)	7.35 (6.41, 8.60)	7.89 (7.14, 8.33)	0.227

Data are presented as median (Q1, Q3) or *n* (%). *P* values are unadjusted and should be interpreted descriptively. Continuous variables were compared using the Mann–Whitney U test; categorical variables were compared using the Pearson chi-square test or Fisher exact test, as appropriate. The *P* value for the upper gastrointestinal modifier is a global comparison of its three categories. The complete baseline comparison is provided in Supplementary Table S3. ALT, alanine aminotransferase; BMI, body mass index; CRP, C-reactive protein; IFX, infliximab; PCDAI, Pediatric Crohn's Disease Activity Index; SES-CD, Simple Endoscopic Score for Crohn's Disease; WBC, white blood cell count.

The distribution of the upper gastrointestinal Paris modifier differed between groups (global *P* = 0.004), mainly because L4b was present in 4 of 21 children with PNR (19.0%) and 1 of 95 responders (1.1%). Because this comparison was based on only five L4b cases, it was considered a sparse exploratory signal. No clear differences were observed in PCDAI, SES-CD, C-reactive protein, albumin, prealbumin, infliximab dose, or concomitant immunomodulator use.

### Association between low baseline ALT and PNR

Individual ALT values and the rounded working threshold are displayed in Figure 1A. In unadjusted Firth logistic regression, low ALT was associated with PNR (OR, 3.78; 95% CI, 1.40–11.71; *P* = 0.008). In the primary model adjusted for baseline SES-CD, actual IFX dose in mg/kg, and concomitant immunomodulator use, low ALT remained associated with PNR (OR, 4.03; 95% CI, 1.48–12.65; *P* = 0.006; Table 3).

**Figure 1:**
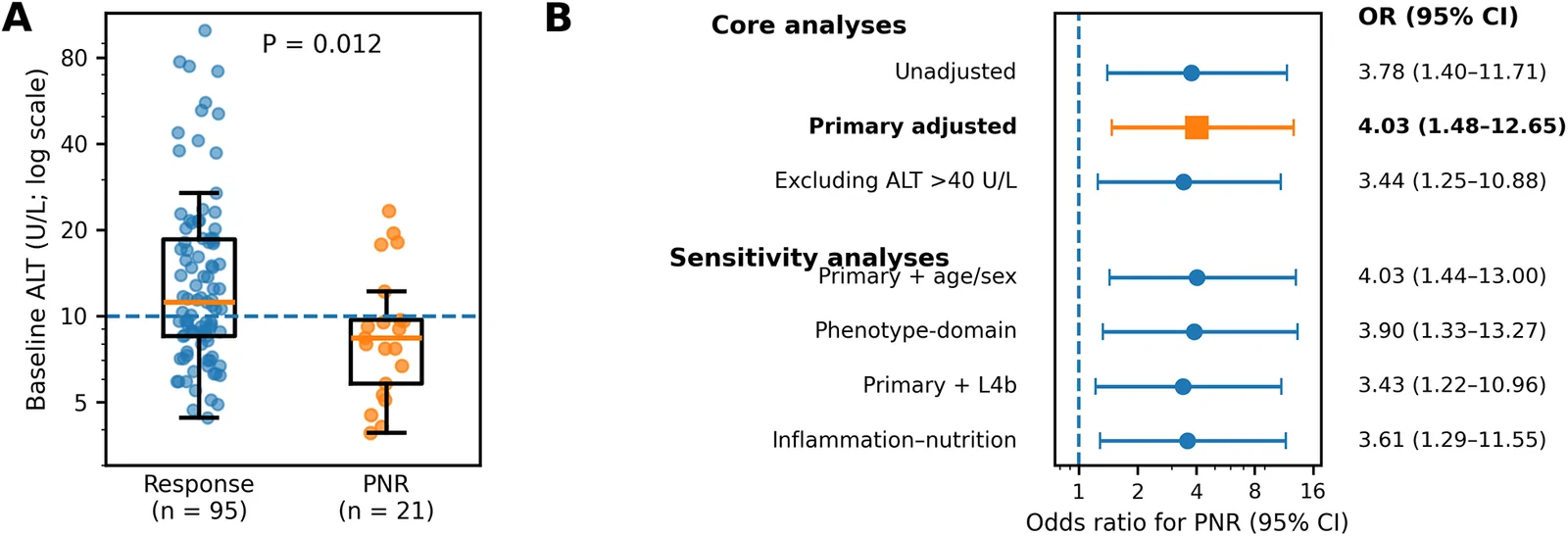
Baseline alanine aminotransferase distribution and stability of its association with primary non-response to infliximab. **(A)** Distribution of baseline ALT in children who responded to infliximab induction and those with primary non-response. Individual observations are overlaid on box plots; boxes indicate the interquartile range, center lines indicate medians, and whiskers extend to 1.5 times the interquartile range. The *y*-axis is logarithmic. The horizontal dashed line indicates the rounded, data-informed working threshold of 10 U/L. Groups were compared using the Mann–Whitney U test. **(B)** Forest plot of the association between low baseline ALT (<10 U/L) and primary non-response across the unadjusted analysis and Firth logistic regression models. The square denotes the primary adjusted model; circles denote the other analyses. Points represent ORs and horizontal lines represent profile penalized-likelihood 95% CIs. The primary model adjusted for baseline SES-CD, actual IFX dose in mg/kg, and concomitant immunomodulator use. The primary + age/sex model additionally adjusted for age and sex. ALT, alanine aminotransferase; CI, confidence interval; IFX, infliximab; OR, odds ratio; PNR, primary non-response; SES-CD, Simple Endoscopic Score for Crohn's Disease.

**Table 3:** Association of baseline ALT with primary non-response to infliximab.

Analysis	Exposure	N	PNR events	Adjustment	OR (95% CI)	*P* value
Unadjusted Firth association	Low ALT (<10 U/L)	116	21	None	3.78 (1.40–11.71)	0.008
Primary adjusted Firth model	Low ALT (<10 U/L)	116	21	Baseline SES-CD, actual IFX dose (mg/kg), and concomitant immunomodulator use	4.03 (1.48–12.65)	0.006
Continuous ALT sensitivity analysis	ALT per 5-U/L decrease	116	21	Same covariates as the primary model	1.50 (1.04–2.59)	0.022
High-ALT exclusion sensitivity analysis	Low ALT (<10 U/L)	107	21	Same covariates as the primary model; 9 patients with ALT >40 U/L excluded	3.44 (1.25–10.88)	0.016

ORs, profile penalized-likelihood 95% CIs, and penalized likelihood-ratio *P* values were obtained from Firth logistic regression. The primary model adjusted for baseline SES-CD, actual IFX dose in mg/kg, and concomitant immunomodulator use. Covariates were selected on clinical grounds rather than by univariable *P*-value screening. ALT <10 U/L was a rounded, data-informed working threshold and is not an externally validated clinical decision boundary. The continuous ALT analysis reports the OR for PNR per 5-U/L decrease. The high-ALT exclusion analysis omitted 9 patients with ALT >40 U/L and retained all 21 PNR events. ALT, alanine aminotransferase; CI, confidence interval; IFX, infliximab; OR, odds ratio; PNR, primary non-response; SES-CD, Simple Endoscopic Score for Crohn's Disease.

When ALT was analyzed continuously, each 5-U/L decrease was associated with higher odds of PNR (OR, 1.50; 95% CI, 1.04–2.59; *P* = 0.022). After exclusion of the nine children with ALT >40 U/L, all of whom were responders, the association for low ALT remained (OR, 3.44; 95% CI, 1.25–10.88; *P* = 0.016).

The low-ALT estimate remained directionally consistent when age and sex were added to the primary model (OR, 4.03; 95% CI, 1.44–13.00), in the phenotype-domain model (OR, 3.90; 95% CI, 1.33–13.27), after adding L4b to the primary covariates (OR, 3.43; 95% CI, 1.22–10.96), and in the inflammation-nutrition model (OR, 3.61; 95% CI, 1.29–11.55; Supplementary Table S1 and Figure 1B). Conventional maximum-likelihood logistic regression yielded a comparable primary estimate (OR, 4.47; profile-likelihood 95% CI, 1.57–14.98). Across 2,000 bootstrap resamples, the median adjusted low-ALT OR was 4.33, the 95% percentile range was 1.55–17.22, and 99.7% of estimates were >1. These analyses preserved the direction of association but also demonstrated substantial uncertainty related to the limited number of PNR events.

### Exploratory ROC analysis

Exploratory receiver operating characteristic analysis showed limited discrimination of continuous ALT for PNR (AUC, 0.677; 2,000-replicate stratified bootstrap 95% CI, 0.547–0.802; Supplementary Figure S1). The pROC Youden threshold was 9.9 U/L, with sensitivity of 76.2% and specificity of 55.8%; the rounded rule of ALT <10 U/L produced the same operating point in this cohort. Across 5,000 nonparametric bootstrap resamples, the median empirical threshold was 9.65 U/L, but the 95% percentile range was wide (5.4–18.2 U/L), and only 52.8% of thresholds were between 9 and 11 U/L. Thus, the ROC analysis contextualized the rounded working threshold but did not establish a stable clinical decision boundary.

## Discussion

In this single-center retrospective cohort of children with Crohn's disease receiving infliximab induction, low baseline alanine aminotransferase was common and identified a subgroup with less favorable inflammation- and nutrition-related laboratory features. Low ALT was also associated with clinically adjudicated primary non-response after adjustment for baseline endoscopic severity, actual infliximab dose, and concomitant immunomodulator use. The direction of association was consistent across the reported sensitivity analyses. These findings are hypothesis-generating and do not establish causality, validated prediction, or a basis for treatment selection.

ALT is conventionally interpreted as a marker of hepatocellular injury when elevated, whereas the clinical meaning of a low value is less familiar. Studies in older adults and chronic disease populations have linked low ALT with frailty, sarcopenia, and adverse outcomes (8–14). Evidence in IBD is more limited but broadly consistent. A nationwide adult study associated low ALT with greater inflammatory activity and lower hemoglobin and albumin levels (15), and a pediatric IBD cohort reported associations between low transaminase levels and unfavorable nutritional and clinical characteristics (16). The present study extends these observations to children with CD undergoing IFX induction and relates the low-ALT phenotype to an early treatment outcome.

The accompanying laboratory phenotype is important for interpretation. Children with low ALT had lower hemoglobin, albumin, and prealbumin levels and higher C-reactive protein and erythrocyte sedimentation rate, whereas PCDAI and SES-CD showed no clear between-group differences. No clear between-group difference in BMI was observed either by ALT status (*P* = 0.903) or by response status (*P* = 0.132). Pediatric CD may involve reduced intake, chronic systemic inflammation, altered body composition, muscle depletion, and impaired growth (17–20). Importantly, BMI does not distinguish lean from fat mass; recent pediatric IBD data show that substantial lean-mass deficits can be present despite a normal BMI Z-score (27). Thus, the absence of a clear BMI difference does not exclude altered body composition. Low ALT may therefore accompany aspects of nutritional reserve and systemic disease burden that are not fully represented by symptom-based, endoscopic, or conventional anthropometric measures. Nevertheless, ALT is not a direct measure of muscle mass, dietary intake, or protein-energy status, and the present data cannot identify which component of this phenotype is most relevant.

A possible relationship with IFX response may involve host factors that also influence drug exposure. Pediatric population pharmacokinetic models identify body weight, albumin, erythrocyte sedimentation rate, and anti-IFX antibodies among covariates relevant to IFX exposure, supporting the broader concept that nutritional and inflammatory state can influence pharmacokinetics (28, 29). The 2025 NASPGHAN position paper specifically emphasizes therapeutic drug monitoring as a tool for optimizing biologic dosing and preventing pharmacokinetic failure in pediatric IBD (30). However, IFX trough concentrations and anti-drug antibodies were unavailable in this cohort. Low ALT therefore cannot be interpreted as evidence of a specific pharmacokinetic mechanism, and the present findings do not imply that low ALT itself alters IFX clearance. It may instead be a nonspecific correlate of a host state in which inadequate exposure, persistent inflammation, nutritional depletion, or other unmeasured factors coexist. Prospective studies incorporating therapeutic drug monitoring are needed to distinguish among these possibilities.

The analyses were designed to reduce, not eliminate, problems arising from the small event count. Firth logistic regression was selected because only 21 children had PNR and several phenotype categories were sparse. The adjusted estimate for low ALT was similar to the unadjusted and conventional maximum-likelihood estimates. Across 2,000 bootstrap resamples, the median OR remained close to the primary estimate and 99.7% of estimates exceeded 1, although the percentile range was wide. Modeling ALT continuously produced a consistent result in the full cohort, whereas the estimate became less precise after ALT values >40 U/L were excluded. Firth penalization can reduce finite-sample bias and address separation in logistic regression (26), but it does not add information, remove residual confounding, or fully prevent overfitting. The estimated magnitude of association should therefore be regarded as imprecise and hypothesis-generating.

The 10-U/L definition also requires cautious interpretation. It was a rounded, data-informed working threshold rather than a prespecified pediatric reference limit. The full-cohort pROC Youden threshold was 9.9 U/L, but ALT showed only limited discrimination for PNR (AUC, 0.677), and the bootstrap distribution of empirical thresholds was broad: the median was 9.65 U/L, the 95% percentile range was 5.4–18.2 U/L, and only 52.8% fell between 9 and 11 U/L. The proximity of the full-cohort threshold to 10 U/L therefore does not confirm a stable biological boundary. Pediatric reference-interval studies, including data from healthy Han Chinese children, demonstrate age- and sex-related variation in ALT (31). Future studies should distinguish a prognostic working threshold from age-, sex-, and population-specific pediatric ALT reference limits rather than applying a universal lower threshold across childhood. Accordingly, the 10-U/L threshold should be viewed only as a practical cohort-classification rule requiring external validation, not as a pediatric lower reference limit or treatment-decision boundary.

The L4b imbalance also warrants cautious interpretation. Upper gastrointestinal or proximal small-bowel involvement in pediatric CD has been associated in some cohorts with younger onset, greater disease activity, and the need for more aggressive therapy, but its relationship with long-term adverse outcomes remains inconsistent (32, 33). In the present cohort, only five children had L4b disease, so the observed difference may reflect sparse-data instability rather than a reproducible prognostic effect. Because IFX is administered intravenously, impaired intestinal drug absorption is not a plausible explanation; if the signal is real, L4b may instead mark more extensive inflammatory burden or other host factors relevant to treatment response.

This study has several strengths. It evaluated a routinely available biochemical measure in a clinically characterized pediatric CD cohort receiving a relatively consistent IFX induction regimen. PNR was independently adjudicated by two senior pediatric gastroenterologists blinded to baseline ALT and required concurrent clinical, biochemical, and objective endoscopic or cross-sectional imaging evidence. The primary model was deliberately parsimonious and adjusted for clinically relevant endoscopic and treatment-exposure factors rather than selecting covariates through univariable screening. Robustness was examined through continuous-exposure analysis, alternative clinical-domain models, exclusion of high ALT values, comparison with conventional logistic regression, and bootstrap resampling. Displaying individual ALT values also makes the substantial overlap between responders and non-responders transparent.

Several limitations are important. First, the retrospective single-center design limits generalizability and is susceptible to selection bias, measurement variation, and unmeasured confounding. The analytical database contained only eligible included patients; because a contemporaneous screening log was unavailable, the total number initially assessed, detailed exclusion counts, and characteristics of nonincluded patients could not be determined. Requiring an evaluable week-14 assessment may also have excluded some early severe failures. Second, although the structured PNR algorithm required concurrent clinical non-response, biochemical activity, and objective endoscopic or imaging evidence and used blinded dual-reviewer adjudication, it remains a locally operationalized composite that has not been externally validated; outcome misclassification and center-specific practice patterns remain possible. Third, only 21 PNR events were available, resulting in wide CIs and restricting the number and complexity of adjustment variables; sparse findings such as L4b involvement should not be considered established prognostic factors. Fourth, although BMI was available and no clear difference was observed by ALT group or response status, BMI cannot distinguish lean from fat mass; therapeutic drug monitoring, anti-drug antibodies, direct measures of muscle mass or body composition, detailed dietary intake, and standardized nutritional assessment were unavailable. Fifth, ALT was measured only at baseline; dynamic changes, secondary loss of response, mucosal healing, growth, and longer-term outcomes were not evaluated. Finally, both the working threshold and the association were assessed in the same cohort and have not been externally validated.

The immediate clinical implication is limited. A low baseline ALT value may prompt closer review of inflammatory activity, nutritional status, growth, and other factors relevant to treatment response, but it should not independently trigger IFX dose intensification, treatment switching, or other management decisions. Whether a similar association exists for adalimumab or non-TNF biologic and targeted therapies remains unknown and should be evaluated in dedicated agent-specific pediatric cohorts. Multicenter prospective studies should use standardized outcome definitions, prespecified age- and sex-sensitive ALT analyses, therapeutic drug monitoring, and direct body-composition assessment. These studies are needed to determine whether low ALT adds information beyond established clinical, endoscopic, nutritional, and pharmacokinetic measures.

## Conclusion

In children with Crohn's disease receiving infliximab induction, low baseline ALT was associated with an unfavorable inflammation- and nutrition-related laboratory phenotype and with clinically adjudicated primary non-response after adjustment for selected endoscopic and treatment-related factors. This exploratory association and the 10-U/L working threshold require external validation. Low ALT should be considered a potential complementary phenotypic marker, not a stand-alone predictor or treatment guide.

## Data Availability

The datasets presented in this article are not readily available because the dataset contains clinical information from pediatric patients and cannot be publicly shared due to patient privacy protection and ethical restrictions. Access to the dataset is restricted to protect confidentiality and requires appropriate approval from the corresponding institution and ethics committee. Requests to access the datasets should be directed to Jie Wu, wujiedoc@163.com.
