# Supplementary material for: Low baseline alanine aminotransferase is associated with primary non-response to infliximab induction in pediatric Crohn's disease: a retrospective cohort study
Source: Front Pediatr. 2026 Sep 25;14:1948999. doi: 10.3389/fped.2026.1948999 (PMC13649988; doi:10.3389/fped.2026.1948999)
Supplement: Supplementary file 1 [file Supplementaryfile1.docx]

**Supplementary Material**

# Supplementary Methods

**Exposure definition.** Baseline alanine aminotransferase (ALT) was defined using the laboratory value closest to the first infliximab infusion within the preceding 4 weeks. ALT <10 U/L was used as a rounded, data-informed working threshold. The threshold was informed by an exploratory receiver operating characteristic analysis in the study cohort, which yielded a Youden threshold of 9.9 U/L, together with clinical interpretability and prior inflammatory bowel disease literature. It was not treated as a prespecified pediatric reference limit or a validated treatment-decision boundary.

**Primary and sensitivity regression analyses.** Analyses were conducted using R version 4.5.2. Firth logistic regression was fitted with the logistf package because only 21 children had primary non-response and several phenotype categories were sparse. The primary model included low ALT, baseline Simple Endoscopic Score for Crohn's Disease, actual infliximab dose in mg/kg, and concomitant immunomodulator use. Sensitivity analyses modeled ALT continuously per 5-U/L decrease; repeated the primary model after excluding ALT values >40 U/L; added age and sex to the primary covariates; evaluated phenotype-domain, L4b-extended, and inflammation-nutrition models; and refitted the primary model using conventional maximum-likelihood logistic regression. Variables required for all models were complete, and no missing-data imputation was performed.

**Bootstrap stability analyses.** Internal stability of the low-ALT coefficient from the primary Firth model was evaluated using 2,000 ordinary nonparametric bootstrap resamples of the full cohort. The low-ALT odds ratio was extracted from each resample. Results were summarized using the median, 2.5th and 97.5th percentiles, and the proportion of estimates greater than 1. These summaries describe internal estimate variability and do not constitute external validation or remove residual confounding.

**Exploratory receiver operating characteristic analyses.** Continuous ALT discrimination for primary non-response was evaluated with the pROC package. The area under the receiver operating characteristic curve was reported with a 2,000-replicate stratified bootstrap 95% confidence interval. The full-cohort Youden threshold, sensitivity, and specificity were calculated. Threshold stability was examined separately using 5,000 ordinary nonparametric bootstrap resamples, with the Youden threshold recalculated in each resample. The broad threshold distribution was interpreted as evidence that approximately 10 U/L is a practical working threshold rather than a stable biological boundary.

**Supplementary Table S1.** Sensitivity, resampling, and cutoff-stability analyses

| **Analysis** | **Exposure or measure** | **N / resamples** | **PNR events** | **Adjustment or method** | **Estimate or result** |
| --- | --- | --- | --- | --- | --- |
| **Panel A. Sensitivity analyses of the low-ALT association** | | | | | |
| Primary model additionally adjusted for age and sex | Low ALT (<10 U/L) | 116 | 21 | Primary-model covariates plus age and sex | 4.03 (1.44–13.00) |
| Phenotype-domain Firth model | Low ALT (<10 U/L) | 116 | 21 | L4b involvement, complicated behavior, and perianal disease | 3.90 (1.33–13.27) |
| Primary Firth model additionally adjusted for L4b | Low ALT (<10 U/L) | 116 | 21 | Primary-model covariates plus L4b involvement | 3.43 (1.22–10.96) |
| Inflammation–nutrition-domain Firth model | Low ALT (<10 U/L) | 116 | 21 | SES-CD, CRP, and albumin | 3.61 (1.29–11.55) |
| Continuous ALT after excluding ALT >40 U/L | ALT per 5-U/L decrease | 107 | 21 | Same covariates as the primary model | 1.51 (0.99–2.60) |
| Maximum-likelihood primary model | Low ALT (<10 U/L) | 116 | 21 | Same covariates as the primary model; profile-likelihood CI | 4.47 (1.57–14.98) |
| **Panel B. Internal stability and exploratory discrimination** | | | | | |
| Firth coefficient bootstrap | Distribution of the adjusted low-ALT OR | 2,000 | 21 in original cohort | Ordinary nonparametric bootstrap of the full primary model | Median OR, 4.33; 95% percentile range, 1.55–17.22; 99.7% of ORs >1 |
| ROC AUC bootstrap | Continuous ALT discrimination for PNR | 2,000 | 21 in original cohort | Stratified bootstrap using pROC | AUC, 0.677; 95% CI, 0.547–0.802 |
| Empirical Youden cut-point in the full cohort | Continuous ALT | 116 | 21 | pROC Youden index | 9.9 U/L; sensitivity, 76.2%; specificity, 55.8% |
| ROC cut-point bootstrap | Distribution of empirical Youden cut-points | 5,000 | 21 in original cohort | Ordinary nonparametric bootstrap; pROC Youden threshold recalculated | Median, 9.65 U/L; 95% percentile range, 5.4–18.2; 52.8% within 9–11 U/L |

Firth model estimates are odds ratios with profile penalized-likelihood 95% confidence intervals. The age-and-sex sensitivity model retained the primary-model covariates and added age and sex.

L4b involvement was present in only 5 patients (4 with primary non-response); phenotype-domain estimates should be interpreted as sparse sensitivity analyses rather than validated prognostic effects.

Bootstrap percentile ranges describe internal estimate variability and do not constitute external validation or remove residual confounding. The wide cut-point distribution supports interpreting 10 U/L as a working threshold rather than a stable biological boundary.

AUC, area under the receiver operating characteristic curve; ALT, alanine aminotransferase; CI, confidence interval; CRP, C-reactive protein; IFX, infliximab; OR, odds ratio; PNR, primary non-response; ROC, receiver operating characteristic; SES-CD, Simple Endoscopic Score for Crohn's Disease.

**Supplementary Table S2.** Complete baseline characteristics of children with Crohn's disease according to baseline ALT status

| **Variable** | **Total (n = 116)** | **Non-low ALT (n = 58)** | **Low ALT (n = 58)** | **P value** |
| --- | --- | --- | --- | --- |
| **Demographic and anthropometric characteristics** | | | | |
| Male sex, n (%) | 72 (62.1) | 40 (69.0) | 32 (55.2) | 0.126 |
| Age, years | 12.0 (9.0, 13.5) | 11.8 (9.8, 13.5) | 12.1 (8.6, 13.5) | 0.799 |
| Disease duration, months | 6.0 (2.9, 16.0) | 5.5 (2.0, 12.0) | 7.5 (3.0, 18.2) | 0.211 |
| BMI, kg/m² | 15.12 (13.78, 17.21) | 15.09 (13.59, 18.23) | 15.45 (13.90, 17.04) | 0.903 |
| **Disease phenotype and activity** | | | | |
| Paris disease location, n (%) |  |  |  | 0.375 |
| L1 | 8 (6.9) | 5 (8.6) | 3 (5.2) |  |
| L2 | 23 (19.8) | 8 (13.8) | 15 (25.9) |  |
| L3 | 82 (70.7) | 44 (75.9) | 38 (65.5) |  |
| Isolated L4 | 3 (2.6) | 1 (1.7) | 2 (3.4) |  |
| Upper gastrointestinal modifier, n (%) |  |  |  | 0.163 |
| None | 77 (66.4) | 43 (74.1) | 34 (58.6) |  |
| L4a | 34 (29.3) | 14 (24.1) | 20 (34.5) |  |
| L4b | 5 (4.3) | 1 (1.7) | 4 (6.9) |  |
| Disease behavior, n (%) |  |  |  | 0.086 |
| B1 | 77 (66.4) | 35 (60.3) | 42 (72.4) |  |
| B2 | 21 (18.1) | 11 (19.0) | 10 (17.2) |  |
| B3 | 12 (10.3) | 10 (17.2) | 2 (3.4) |  |
| B2B3 | 6 (5.2) | 2 (3.4) | 4 (6.9) |  |
| Perianal disease, n (%) | 23 (19.8) | 8 (13.8) | 15 (25.9) | 0.103 |
| Growth failure, n (%) | 39 (33.6) | 19 (32.8) | 20 (34.5) | 0.844 |
| PCDAI | 30.00 (15.00, 38.12) | 28.50 (15.62, 36.88) | 32.00 (15.62, 40.00) | 0.484 |
| SES-CD | 6.00 (4.00, 11.00) | 6.00 (4.00, 11.00) | 7.00 (4.00, 10.00) | 0.772 |
| **Laboratory characteristics** | | | | |
| CRP, mg/L | 6.50 (4.00, 17.75) | 5.00 (4.00, 9.50) | 8.00 (5.00, 39.00) | 0.019 |
| ESR, mm/h | 21.00 (11.00, 39.25) | 13.50 (7.25, 34.50) | 27.00 (14.25, 43.25) | 0.014 |
| WBC, ×10⁹/L | 7.78 (5.74, 10.29) | 7.07 (5.62, 9.70) | 7.92 (5.94, 10.79) | 0.237 |
| Neutrophils, ×10⁹/L | 4.63 (3.26, 6.12) | 3.99 (3.04, 5.48) | 4.76 (3.74, 6.99) | 0.081 |
| Lymphocytes, ×10⁹/L | 1.94 (1.43, 2.82) | 1.98 (1.54, 2.78) | 1.82 (1.36, 2.91) | 0.693 |
| Monocytes, ×10⁹/L | 0.51 (0.38, 0.83) | 0.47 (0.39, 0.67) | 0.65 (0.34, 0.94) | 0.061 |
| Platelets, ×10⁹/L | 393.50 (287.75, 508.50) | 383.00 (281.50, 460.50) | 410.50 (317.75, 567.75) | 0.121 |
| Hemoglobin, g/L | 108.00 (99.00, 122.00) | 111.00 (102.25, 128.75) | 105.00 (98.00, 114.00) | 0.008 |
| Albumin, g/L | 37.60 (33.77, 40.42) | 38.35 (34.90, 41.50) | 36.30 (32.25, 38.50) | 0.002 |
| Prealbumin, mg/L | 140.00 (107.50, 195.50) | 151.00 (116.25, 215.75) | 127.50 (98.00, 169.50) | 0.007 |
| Fecal calprotectin reporting category, n (%) |  |  |  | 0.496 |
| Measured 0 | 67 (57.8) | 31 (53.4) | 36 (62.1) |  |
| Measured >0 | 16 (13.8) | 10 (17.2) | 6 (10.3) |  |
| Reported >1800 | 33 (28.4) | 17 (29.3) | 16 (27.6) |  |
| **Treatment characteristics** | | | | |
| Prior biologic exposure, n (%) | 7 (6.0) | 3 (5.2) | 4 (6.9) | 1.000 |
| Nutrition support, n (%) |  |  |  | 0.252 |
| Complete enteral nutrition | 73 (62.9) | 33 (56.9) | 40 (69.0) |  |
| Partial enteral nutrition | 11 (9.5) | 6 (10.3) | 5 (8.6) |  |
| CDED | 15 (12.9) | 11 (19.0) | 4 (6.9) |  |
| No nutritional intervention | 17 (14.7) | 8 (13.8) | 9 (15.5) |  |
| Concomitant immunomodulator use, n (%) | 41 (35.3) | 20 (34.5) | 21 (36.2) | 0.846 |
| Concomitant corticosteroid use, n (%) | 29 (25.0) | 14 (24.1) | 15 (25.9) | 0.830 |
| IFX dose, mg/kg | 7.46 (6.48, 8.51) | 7.58 (6.81, 8.65) | 7.26 (6.30, 8.32) | 0.171 |
| **Induction outcome** | | | | |
| Primary non-response to IFX, n (%) | 21 (18.1) | 5 (8.6) | 16 (27.6) | 0.008 |

Data are presented as median (Q1, Q3) or n (%). P values are unadjusted and should be interpreted descriptively.

Continuous variables were compared using the Mann-Whitney U test. Categorical variables were compared using the Pearson chi-square test or Fisher exact test, as appropriate. Sparse multicategory tables were evaluated using a Monte Carlo conditional test based on 200,000 random tables (seed 20260721).

Fecal calprotectin values of 0 were retained as actual measured values. Results reported as >1800 were treated as upper-limit-censored reports and were not assigned an exact numeric value.

Low ALT was defined as baseline ALT <10 U/L. PNR required concurrent clinical non-response, biochemical activity, and objective endoscopic or cross-sectional imaging evidence after infliximab induction.

ALT, alanine aminotransferase; BMI, body mass index; CDED, Crohn's Disease Exclusion Diet; CRP, C-reactive protein; ESR, erythrocyte sedimentation rate; FC, fecal calprotectin; IFX, infliximab; PCDAI, Pediatric Crohn's Disease Activity Index; PNR, primary non-response; SES-CD, Simple Endoscopic Score for Crohn's Disease; WBC, white blood cell count.

**Supplementary Table S3.** Complete baseline characteristics of children with Crohn's disease according to primary response to infliximab induction

| **Variable** | **Total (n = 116)** | **Response (n = 95)** | **Primary non-response (n = 21)** | **P value** |
| --- | --- | --- | --- | --- |
| **Demographic and anthropometric characteristics** | | | | |
| Male sex, n (%) | 72 (62.1) | 59 (62.1) | 13 (61.9) | 0.986 |
| Age, years | 12.0 (9.0, 13.5) | 12.1 (9.8, 13.5) | 11.6 (5.3, 12.6) | 0.101 |
| Disease duration, months | 6.0 (2.9, 16.0) | 6.0 (3.0, 16.0) | 6.0 (2.0, 16.0) | 0.911 |
| BMI, kg/m² | 15.12 (13.78, 17.21) | 15.36 (13.98, 17.45) | 14.53 (12.91, 16.41) | 0.132 |
| **Disease phenotype and activity** | | | | |
| Paris disease location, n (%) |  |  |  | 0.942 |
| L1 | 8 (6.9) | 7 (7.4) | 1 (4.8) |  |
| L2 | 23 (19.8) | 18 (18.9) | 5 (23.8) |  |
| L3 | 82 (70.7) | 68 (71.6) | 14 (66.7) |  |
| Isolated L4 | 3 (2.6) | 2 (2.1) | 1 (4.8) |  |
| Upper gastrointestinal modifier, n (%) |  |  |  | 0.004 |
| None | 77 (66.4) | 65 (68.4) | 12 (57.1) |  |
| L4a | 34 (29.3) | 29 (30.5) | 5 (23.8) |  |
| L4b | 5 (4.3) | 1 (1.1) | 4 (19.0) |  |
| Disease behavior, n (%) |  |  |  | 0.060 |
| B1 | 77 (66.4) | 66 (69.5) | 11 (52.4) |  |
| B2 | 21 (18.1) | 15 (15.8) | 6 (28.6) |  |
| B3 | 12 (10.3) | 11 (11.6) | 1 (4.8) |  |
| B2B3 | 6 (5.2) | 3 (3.2) | 3 (14.3) |  |
| Perianal disease, n (%) | 23 (19.8) | 18 (18.9) | 5 (23.8) | 0.562 |
| Growth failure, n (%) | 39 (33.6) | 30 (31.6) | 9 (42.9) | 0.322 |
| PCDAI | 30.00 (15.00, 38.12) | 30.00 (16.25, 36.25) | 30.00 (15.00, 40.00) | 0.782 |
| SES-CD | 6.00 (4.00, 11.00) | 6.00 (4.00, 11.00) | 6.00 (5.00, 12.00) | 0.675 |
| **Laboratory characteristics** | | | | |
| ALT, U/L | 9.90 (7.70, 18.10) | 11.20 (8.50, 18.50) | 8.40 (5.80, 9.70) | 0.012 |
| Low ALT (<10 U/L), n (%) | 58 (50.0) | 42 (44.2) | 16 (76.2) | 0.008 |
| CRP, mg/L | 6.50 (4.00, 17.75) | 6.00 (4.00, 13.50) | 8.00 (5.00, 39.00) | 0.123 |
| ESR, mm/h | 21.00 (11.00, 39.25) | 21.00 (10.50, 38.00) | 27.00 (13.00, 41.00) | 0.215 |
| WBC, ×10⁹/L | 7.78 (5.74, 10.29) | 7.23 (5.63, 9.13) | 9.89 (6.68, 13.80) | 0.045 |
| Neutrophils, ×10⁹/L | 4.63 (3.26, 6.12) | 4.21 (3.12, 5.72) | 5.42 (4.16, 8.37) | 0.016 |
| Lymphocytes, ×10⁹/L | 1.94 (1.43, 2.82) | 1.92 (1.45, 2.77) | 2.65 (1.20, 3.31) | 0.526 |
| Monocytes, ×10⁹/L | 0.51 (0.38, 0.83) | 0.49 (0.36, 0.78) | 0.72 (0.53, 0.99) | 0.017 |
| Platelets, ×10⁹/L | 393.50 (287.75, 508.50) | 394.00 (287.50, 506.00) | 383.00 (315.00, 525.00) | 0.886 |
| Hemoglobin, g/L | 108.00 (99.00, 122.00) | 108.00 (100.00, 119.00) | 104.00 (98.00, 127.00) | 0.835 |
| Albumin, g/L | 37.60 (33.77, 40.42) | 37.60 (34.20, 40.45) | 38.00 (32.70, 39.10) | 0.680 |
| Prealbumin, mg/L | 140.00 (107.50, 195.50) | 144.00 (108.00, 200.50) | 124.00 (105.00, 195.00) | 0.616 |
| Fecal calprotectin reporting category, n (%) |  |  |  | 0.522 |
| Measured 0 | 67 (57.8) | 57 (60.0) | 10 (47.6) |  |
| Measured >0 | 16 (13.8) | 13 (13.7) | 3 (14.3) |  |
| Reported >1800 | 33 (28.4) | 25 (26.3) | 8 (38.1) |  |
| **Treatment characteristics** | | | | |
| Prior biologic exposure, n (%) | 7 (6.0) | 5 (5.3) | 2 (9.5) | 0.609 |
| Nutrition support, n (%) |  |  |  | 0.501 |
| Complete enteral nutrition | 73 (62.9) | 58 (61.1) | 15 (71.4) |  |
| Partial enteral nutrition | 11 (9.5) | 10 (10.5) | 1 (4.8) |  |
| CDED | 15 (12.9) | 14 (14.7) | 1 (4.8) |  |
| No nutritional intervention | 17 (14.7) | 13 (13.7) | 4 (19.0) |  |
| Concomitant immunomodulator use, n (%) | 41 (35.3) | 34 (35.8) | 7 (33.3) | 0.831 |
| Concomitant corticosteroid use, n (%) | 29 (25.0) | 22 (23.2) | 7 (33.3) | 0.330 |
| IFX dose, mg/kg | 7.46 (6.48, 8.51) | 7.35 (6.41, 8.60) | 7.89 (7.14, 8.33) | 0.227 |

Data are presented as median (Q1, Q3) or n (%). P values are unadjusted and should be interpreted descriptively.

Continuous variables were compared using the Mann-Whitney U test. Categorical variables were compared using the Pearson chi-square test or Fisher exact test, as appropriate. Sparse multicategory tables were evaluated using a Monte Carlo conditional test based on 200,000 random tables (seed 20260721).

Fecal calprotectin values of 0 were retained as actual measured values. Results reported as >1800 were treated as upper-limit-censored reports and were not assigned an exact numeric value.

Low ALT was defined as baseline ALT <10 U/L. PNR required concurrent clinical non-response, biochemical activity, and objective endoscopic or cross-sectional imaging evidence after infliximab induction.

ALT, alanine aminotransferase; BMI, body mass index; CDED, Crohn's Disease Exclusion Diet; CRP, C-reactive protein; ESR, erythrocyte sedimentation rate; FC, fecal calprotectin; IFX, infliximab; PCDAI, Pediatric Crohn's Disease Activity Index; PNR, primary non-response; SES-CD, Simple Endoscopic Score for Crohn's Disease; WBC, white blood cell count.

**Supplementary Figure S1.** Exploratory receiver operating characteristic analysis of baseline alanine aminotransferase for primary non-response to infliximab

Exploratory receiver operating characteristic analysis of baseline alanine aminotransferase for differentiating children with and without primary non-response to infliximab. The area under the curve was 0.677 (2,000-replicate stratified bootstrap 95% CI, 0.547-0.802). The pROC Youden-derived threshold was 9.9 U/L, with sensitivity of 76.2% and specificity of 55.8%. The rounded working threshold of ALT <10 U/L produced the same operating point in this cohort. The dashed diagonal line represents no discrimination. This analysis was exploratory and was not used to establish a validated clinical decision threshold.
